# Hospital architecture matters – rethinking the role of mixed sex wards and family rooms in psychiatric hospitals

**DOI:** 10.1192/j.eurpsy.2023.170

**Published:** 2023-07-19

**Authors:** N. Jovanovic

**Affiliations:** Wolfson Institute of Population Health, Queen Mary University of London, London, United Kingdom

## Abstract

**Abstract:**

Hospital built environment can affect patient clinical outcomes, patient satisfaction with care and treatment, staff performance and wellbeing, and carers/visitors’ engagement with services. Little is known about which urban planning, architecture and interior design characteristics can make environments therapeutic or detrimental for users.

We hope that the audience attending this presentation will i) get a good understanding of the impact of the hospital-built environment on patients, staff and visitors/carers and ii) understand which design elements can improve patient satisfaction with care.

As hospitals are among the most expensive facilities to build, their design should be guided by research evidence. In this presentation, we will review existing research evidence in this field and present our study of 18 psychiatric hospitals in Italy and the United Kingdom. Our findings indicate that out of several hospital built environment characteristics, two have the power to increase patient satisfaction with care.

These are (availability of ) mixed-sex wards and rooms to meet family off wards.

We will show vignettes to further explore the role of mixed-sex wards and family rooms and discuss how to implement them when renovating, adapting or building mental health care facilities.

**Disclosure of Interest:**

None Declared

